# Perspectives of military-affiliated women on lethal means safety: A systematic review

**DOI:** 10.1371/journal.pone.0344104

**Published:** 2026-03-05

**Authors:** Melissa A. Litschi, Megan Lafferty, Amy Riegelman, Steven L. Lancaster, David J. Linkh

**Affiliations:** 1 Institute for Quality, Cohen Veterans Network, Stamford, Connecticut, United States of America; 2 Center to Improve Veteran Involvement in Care (CIVIC), VA Portland Health Care System, Portland, Oregon, United States of America; 3 University Libraries, University of Minnesota, Minneapolis, Minnesota, United States of America; Uniformed Services University of the Health Sciences, UNITED STATES OF AMERICA

## Abstract

**Background:**

Women are the fastest-growing military cohort, with suicide rates rising faster than among veteran men and civilian women. Lethal means include firearms, used more often than by civilian women, and non-firearm methods like poisoning, used more than by veteran men. Despite these trends, most lethal means research is gender-neutral, and clinical guidance lacks gender-informed strategies.

**Objectives:**

To synthesize literature on military-affiliated women’s perspectives on lethal means safety and how it should be addressed in suicide prevention conversations.

**Design:**

Qualitative systematic review.

**Data sources:**

APA PsycINFO via Ovid, Ovid MEDLINE ALL, select government and non-profit websites, and citation searching through December 2024.

**Methods:**

Screening occurred in two stages (title/abstract and full text), with 30% of records double-screened. Quality appraisal was conducted using the Critical Appraisal Skill Programme (CASP), Consolidated Criteria for Reporting Qualitative Research (COREQ) Checklist, and Mixed-Methods Appraisal Tool (MMAT). Qualitative and quantitative data fragments were extracted and organized into domains on ownership, access, and means safety interventions. Thematic synthesis used an inductive coding approach. The protocol was published on OSF (https://doi.org/10.17605/OSF.IO/Z8DJH).

**Results:**

Six of the seven articles included perspectives of VHA-enrolled women veterans on firearms or firearm safety counseling, two included active duty women. Three themes on firearm ownership and access emerged, highlighting variations in: understandings of safety surrounding firearms, the impact of military service and identity on firearm beliefs, and the role of spouses/partners in household firearm access. Three themes relating to lethal means safety counseling were identified: general acceptability of interventions, trust as a critical element of means safety conversations, and spouses as stakeholders in means safety conversations.

**Conclusions:**

Lethal means safety counseling for military-affiliated women must be trauma-sensitive and gender-informed. Future research must expand beyond VA contexts and examine the impact and feasibility of engaging spouses in safety interventions.

## Introduction

Concerning trends in military and veteran suicide have led the White House [1], Department of Defense [2], and Department of Veteran Affairs [3] to identify suicide prevention as a leading clinical and public health priority. Despite this, suicide rates among military-affiliated women are increasing at rates that far outpace both military-affiliated men and non-military affiliated women. From 2020–2021, suicide rates among women veterans spiked 24.1% before returning to prior year levels, compared to a 6.3% rise for veteran men [3–5]. During this same period age-adjusted suicide rates for veteran women were 166.1% higher than for non-veteran women, while the difference between veteran and non-veteran men was only 43.4% [5]. Further, suicide was identified as the second leading cause of death among active duty women service members between 2015 and 2019 [6]. Women are the fastest growing military cohort, with 2.5 million women who have served or are currently serving [7,8]. As women continue to join the military at increasing rates, suicide prevention for military-affiliated women warrants further attention.

Women veterans and service members are subject to unique, interconnected experiences that may contribute to suicide risk due to their gender, military identities, and trauma exposures, including military sexual trauma (MST) and intimate partner violence (IPV) [9–11]. For example, military-affiliated women experience increased suicidal ideation and behaviors compared to military-affiliated men [12] and elevated suicide risk during deployment [13]. Further, premilitary and military sexual violence is associated with suicide attempts among veteran women [14], while MST-related PTSD is over three times more likely to be associated with suicidal ideation compared to combat/deployment-related PTSD [15].

Lethal means restriction is an evidence-based approach to suicide prevention [16], and lethal means safety counseling (LMSC) interventions have been shown to result in safe storage behavior changes [17]. However, current best-practices in LMSC have taken a gender-neutral approach which does not consider the gender-specific needs and preferences of women [11,18–20]. Patterns in lethal means selection among military-affiliated women who die by suicide differ drastically when compared to both non-military affiliated women and military-affiliated men. Approximately 50% of suicide deaths among veteran women involve firearms, a rate 281.1% higher than among non-veteran women in 2021 [5]. Conversely, the other 50% of veteran women use non-firearm means, including asphyxiation and poisoning, at higher rates than veteran men [5].

Persistent misconceptions among clinicians about the way women, particularly women who have served in the armed forces, engage in suicide behaviors may lead to incomplete firearm risk assessments and overlooked opportunities for LMSC. For example, firearm screening often focuses on *ownership,* but women veterans have higher rates of household access to firearms they do not personally own [21,22] and may not report when queried about ownership [23]. Additionally, current best-practices do not address the impacts of MST and IPV on firearms safety discussions, both of which are notable suicide risk factors among veteran women [14,24–26]. Furthermore, clinical guidance for non-firearms means is lacking, disproportionately impacting military-affiliated women [23].

Firearms related LMSC has been identified as a priority within suicide prevention efforts for service members and veterans due to the high rates of firearm suicides (over 70%) [2,3]. However, there remains a limited understanding of women’s perspectives on firearms and LMSC and therefore an absence of tailored approaches for military-affiliated women in clinical interventions [19,20,23]. The effectiveness of LMSC may be limited without addressing stakeholder perspectives and lived experiences. For example, there remains a lack of clinical guidance for navigating means safety in the context of IPV [27]. There is an urgent need to identify and adapt best practices for this rapidly growing and high-risk population.

The aim of this review is to synthesize existing literature on military-affiliated women’s perspectives on lethal means safety and how this topic should be addressed in suicide prevention conversations, such as LMSC. In this systematic review we ask: what are the perspectives and preferences of military-affiliated women regarding the safe storage, use, access, and ownership of firearms and other lethal means, including how these topics should be addressed in suicide prevention efforts?

## Methods

In this systematic review, we included quantitative, qualitative, and mixed-methods studies which specifically examined the perspectives and preferences of military-affiliated women in relation to our research question outlined above. Our thematic synthesis was guided by recommendations from Thomas and Harden [28] and reporting followed PRISMA [29] and ENTREQ [30] guidelines. The protocol for this review was published on OSF (https://doi.org/10.17605/OSF.IO/Z8DJH).

### Search strategy

#### Information sources.

To target the relevant literature on the research questions, term harvesting and benchmarking were used to ensure discovery. Using a set of six relevant and nearly relevant studies, we investigated which databases and aggregators regularly indexed the literature on the present topic. APA PsycINFO via Ovid and Ovid MEDLINE ALL were found to include the relevant literature with few exceptions due to irregularly indexed grey literature.

#### Search.

The aforementioned seminal article set was used to investigate existing terms and subject headings. Search strategies focused on a variety of search terms and subject headings representing military affiliated women, lethal means, and perspectives. S1 Appendix contains the full reproducible electronic search strategy. Title, abstract, author-supplied keyword, and subject heading metadata fields were searched with appropriate AND and OR Boolean operators. Database searches were last updated on December 23, 2024. Records were exported from the databases and imported into Covidence, a systematic review web application, and deduplicated. Irregularly indexed records were identified via handsearching and citation searching of included studies [31]. Handsearching on December 23, 2024 targeted reports from Rand (Rand.org), DoD (Defense.gov), VA (VA.gov), and CDC (CDC.gov). Forward citation searching was conducted via Google Scholar on March 31, 2025 while backward citation searching focused on the references lists of included studies. All relevant items not found via the electronic search were added to the corpus of the results.

### Citation screening

#### Inclusion and exclusion criteria.

Included studies examined the perspectives and preferences of military-affiliated women on the safe storage, access, and ownership of lethal means for suicide, including how these topics are addressed in suicide prevention conversations. The target population of military-affiliated women includes US military veterans and service members, including the National Guard and Reserves. Women were defined as individuals whose sex assigned at birth was female and/or who identify as women. Lethal means were defined as items that could be used to attempt or die by suicide, including but not limited to firearms, drugs or toxins, ligatures, sharp or pointed objects, and heights. Given the prevalence of firearm suicide in military populations, articles discussing firearms outside of the context of suicide prevention were eligible for inclusion only if the study fit all other inclusion criteria. Studies were excluded if they did not include military-affiliated women or did not separately report data and analyses relevant to military-affiliated women. Additionally, articles summarizing third-party impressions (e.g., spouses, family members, providers, crisis responders) of the perspectives of military-affiliated women were excluded. Further, articles were excluded if they did not include perspectives and preferences relating to lethal means and/or lethal means safety, if they did not present primary data and related analyses, or if they were epidemiological, electronic health record, national death index, or prevalence studies.

#### Screening process.

All articles identified by the search strategy were uploaded into Covidence. Duplicates were automatically removed by Covidence and manually during the screening process. Screening was completed by two reviewers (MAL and ML) in two separate stages. Screening instructions were provided to both reviewers that included the rationale for the review, research question, inclusion/exclusion criteria, and guiding questions. Both reviewers familiarized themselves with the screening instructions and completed a normalization exercise using a holdout sample of 30 titles and abstracts. Discrepancies were discussed until both reviewers agreed on the rationale for advancing articles to the full text screen or excluding based on the title/abstract. Studies were screened in two stages, title/abstract and full text. Prior to the full text screen, a normalization exercise was completed using two full text articles selected from the holdout sample (one quantitative and one qualitative article) and an exclusion hierarchy was developed. Inter-rater reliability was assessed for each stage using a 30% sample independently coded by two reviewers, and an inter-rater reliability statistic (Cohen’s κ) was calculated by Covidence. Notably, Cohen’s κ is impacted by a high prevalence in one category [32], in this case, the agreement to exclude. Inter-rater agreement was high in both stages, with 13 (1.2%) discrepancies among 1100 double screened titles/abstracts (κ = 0.60) and complete agreement for the 19 double screened full text articles (κ = 1).

### Quality appraisal

Quality appraisal was conducted by authors with relevant expertise using three assessments. The Critical Appraisal Skills Programme (CASP) contains 10 questions across three sections, evaluating methodological validity, analytical rigor, and value of the results [33]. The Consolidated Criteria for Reporting Qualitative Research (COREQ) Checklist contains 32 questions across three domains, research team and reflexivity, study design, and analysis and findings [34]. Qualitative studies were appraised using both the CASP and COREQ checklist (ML/MAL). Mixed-methods and quantitative studies were appraised using the Mixed Methods Appraisal Tool (MMAT; SL/DL) [35]. The MMAT contains two questions relating to clarity of research questions and adequacy of data collection methods which are used to screen all studies as well as sections for different types of publications. The section for quantitative descriptive studies contains five questions relating to sampling strategy, measures, nonresponse bias, and appropriateness of statistical analysis. The section for mixed-methods studies contains five questions relating to study design, integration of findings, and appropriateness of qualitative and quantitative methodologies. Quality appraisals (Table 1) were used to inform our confidence in study results rather than informing inclusion in the review.

**Table 1:** Summary of quality appraisals.

	CASP (10 Total)	COREQ (32 Total)	MMAT (7 Total)
Bond et al., 2024 [36]	–	–	6
Monteith et al., 2020 [37]	10	22	–
Monteith et al., 2023 [26]	–	–	7
Polzer, Holliday, et al., 2023 [38]	9	23	–
Polzer, Rohs, et al., 2023 [27]	9	24	–
Sadler et al., 2020 [39]	–	–	6
Valenstein et al., 2018 [40]	–	–	3

Note: maximum score for each appraisal tool is provided in the heading.

### Data extraction and analysis

Thematic synthesis [28] was utilized to analyze data and analytical fragments across seven included papers. Quantitative data from mixed methods and quantitative studies was treated as qualitative for the purposes of this analysis, with findings indicating shared experiences or perspectives among participants.

Data extraction tables were created for this project corresponding to various elements of the research question, including sections relating to firearm ownership and access, firearm use, firearm storage, access and storage of non-firearm means, lethal means safety messengers, and lethal means safety interventions. Additionally, data was collected relating to study design and participant characteristics (e.g., research questions, sample size and demographics, inclusion/exclusion criteria), as well as analytical fragments presented in the discussion (e.g., future research directions and clinical implications). Authors MAL and ML piloted the extraction table using two articles from the holdout sample, one quantitative article and one qualitative article. Extraction tables were reviewed by both authors to assess agreement in extraction approaches. The first author completed data extraction for the remaining articles. Extracted qualitative data fragments used direct text from the articles and included names of themes and sub-themes, descriptions of each theme and sub-theme, and individual examples and quotes supporting each theme. Quantitative data fragments included prevalence data relating to beliefs, preferences, and firearm practices, as well as relevant statistical associations between certain beliefs, behaviors, or preferences and demographic characteristics and trauma experiences. Extracted data fragments were organized into domains including firearm access and ownership, firearm storage, means safety messengers, and means safety interventions. Extracted data was inductively coded, and codes were organized into themes based on patterns within and across domains. Themes and related codes were reviewed and discussed by the research team and revised until all members reached agreement.

## Results

### Characteristics of included studies

**Fig 1:**
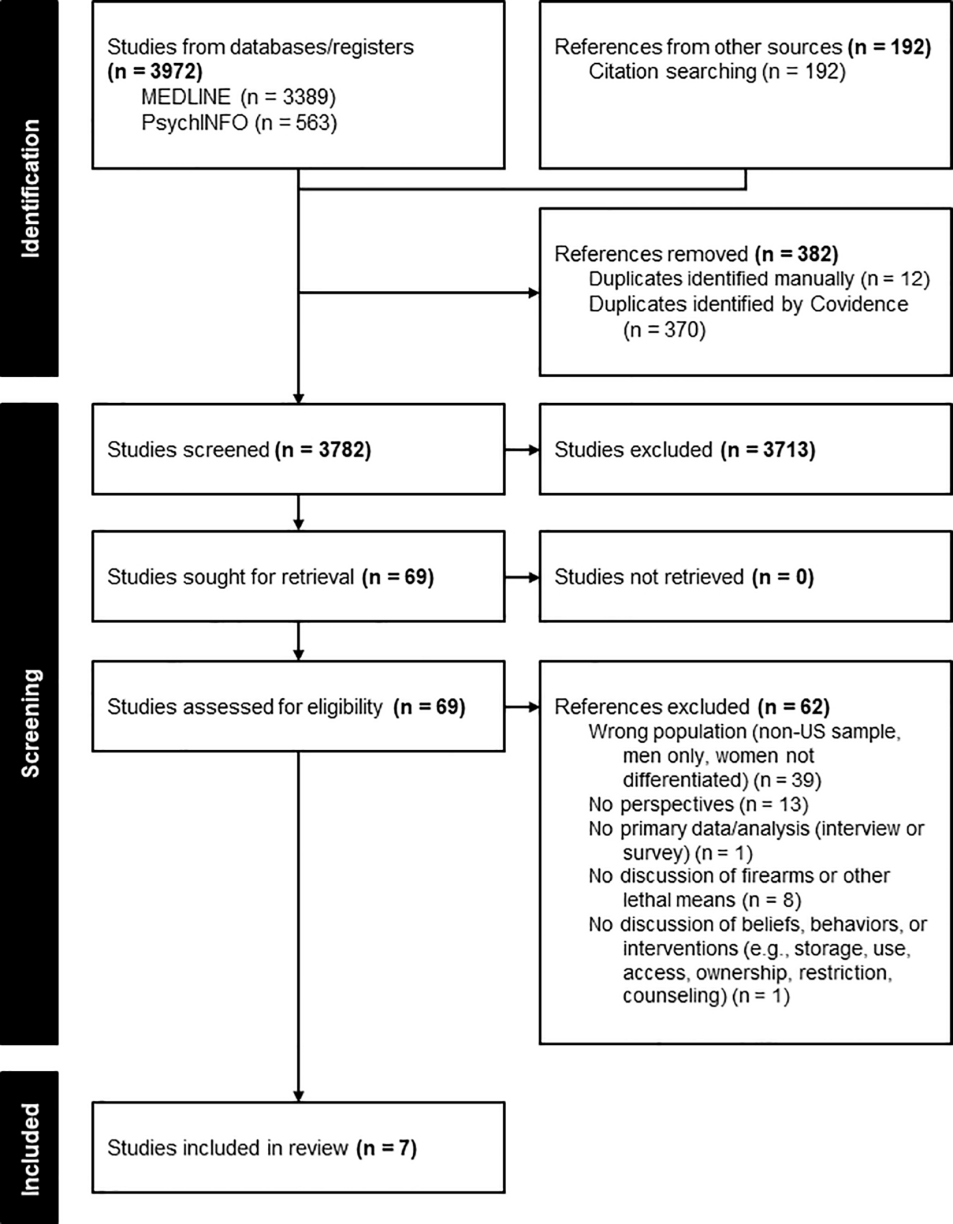
PRISMA flow diagram for identification, screening, and inclusion.

The total results from the search are depicted in Fig 1. PRISMA Flow Diagram. A total of 4164 total records were deduplicated resulting in 3782 records being screened in the first screening stage. Seven articles (Table 2), all published since 2018, were identified for inclusion, including three qualitative articles arising from two studies, three quantitative studies, and one mixed methods study. Two articles included mixed sex samples but reported some of the findings by sex. Only findings specific to female participants were extracted and included in this analysis. Sample sizes of included articles varied widely, including relatively small sample sizes in qualitative and mixed-sex quantitative studies. Any frequencies or percentages reported in the thematic synthesis are intended to provide context for synthesized themes and should be interpreted within the methodological and analytical contexts of their own studies.

All articles focused on perspectives relating to firearms, with no research relating to other means identified. Research largely focused on women veterans eligible or enrolled in VA care, with two quantitative articles whose samples were entirely or predominantly active duty service members. Notably, research presented in six of the articles was conducted within the VA, including research staff, funding, and participants. Research questions across these seven articles were diverse, capturing perspectives on personally owned firearms, household firearms, means safety messengers, and means safety interventions.

**Table 2:** Characteristics of included studies.

Paper	Topic	Participants	Sample Size (% women)	Context	Methods	Select Findings
Bond et al., 2024 [36]^a^	Examination of credible sources for firearm safety messages	US service members who owned firearms	710 (16.6%) 118 women	National sample (Ipsos Knowledge Panel)	Quantitative survey	Service members, law enforcement, and veterans were ranked as the most credible sources for firearm safety messages, including by white women. Rankings varied among sub-populations, with Black women identifying medical professionals and suicide prevention organizations as the most credible sources.
Monteith et al., 2020 [37]^b^	Perspectives and experiences relating to firearms	Female veterans eligible to receive VHA care and currently or previously living in a household with firearms	16 (100%)	VHA sample (Mountain West)	Qualitative interviews	Firearm experiences were facilitated by men before, during, and after military service. Perceptions of firearms as protective tools grew during military service and motivated ownership following service. Trust identified as a critical element of LMSC.
Monteith et al., 2023 [26]^b^	Changes in firearm beliefs and behaviors during the COVID-19 pandemic	Previously deployed post-9/11 women veterans enrolled in VHA services	501 (100%)	VHA sample	Mixed methods survey	Changes in firearm beliefs (14%) and behaviors (22%) reported during the pandemic linked to perceived increased needs to protect self, family, and property and associated with MST history and PTSD symptom severity.
Polzer, Holliday, et al., 2023 [38]^b,c^	Lethal means counseling perspectives, experiences, and preferences	Women veterans with a lifetime history of SI or suicide attempt, post-service history of personal or household firearm access, and prior VHA use	40 (100%)	VHA sample (national enrollment)	Qualitative interviews	Firearm and LMC perspectives shaped by military service history and identity, MST, family, rurality, and experiences with suicidal ideation and attempts. Positive LMC experiences marked by trust, care, and clear communication, and preferences for future LMC included framing discussions around safety and addressing any disclosure-related fears.
Polzer, Rohs, et al., 2023 [27]^b,c^	Perspectives and experiences navigating household firearm access and storage decisions with partners; perspectives on partner involvement in LMC	Women veterans with a lifetime history of SI or suicide attempt, post-service history of personal or household firearm access, and prior VHA use	40 (100%)	VHA sample (national enrollment)	Qualitative interviews	Identified three relational types that impacted firearm storage discussions: collaborative, deferential, and devalued.
Sadler et al., 2020 [39]^b^	Post-deployment experiences and factors associated with keeping weapons nearby for safety	OEF/OIF-era female service members and veterans	978 (100%)	Selected States based on Army/Air Force enrollment location or current residence (Illinois, Iowa, Kansas, Missouri, or Nebraska)	Quantitative survey	One-fifth of participants kept weapons nearby for safety. This behavior was more likely among those who were younger, experienced combat or gender-based trauma, mental health conditions, relied on others for housing, or patrolled their homes.
Valenstein et al., 2018 [40]^a,b^	Acceptability of various firearm safety interventions	Veterans receiving VHA mental health or substance use care in the past 12 months	660 (12.5%) 83 women	VHA sample (Pacific, South Central, South Atlantic, West Mountain South, and West Mountain North)	Quantitative survey	A majority of participant supported health systems level firearm access interventions (93%) and for interventions that substantially limited access (75%, 85% among women). Endorsement of intervention participation among those with household firearms was lower compared to the full sample (50%).

^a^selected findings include the full sample, findings specific to women are identified

^b^study supported by VA funding

^c^data collected as a part of the same study

### Thematic synthesis

Included articles covered a diverse range of research questions relating to perspectives on firearms in the home and preferences relating to firearm safety discussions in the context of mental health and suicide prevention. Six themes were identified across the included articles: (1) women weigh safety considerations when making decisions about firearm ownership, access, and storage, (2) military experiences and identity changed relationships with firearms, (3) relationship dynamics can impact firearm access and storage decisions, (4) lethal means safety interventions are generally perceived as effective and acceptable, but barriers to care remain, (5) trust, caring, and established relationships were critical aspects of accepting LMSC, (6) spouses are key stakeholders in household firearm safety, but relationship dynamics influence their inclusion in care. The first three themes, arising from five articles, related to perspectives and preferences of military-affiliated women regarding the safe storage, use, access, and ownership of firearms and other lethal means. These themes spoke to the reasons why (or why not) women veterans owned firearms, factors that impact their storage decisions, the ways in which they are exposed to firearms they do not own, and barriers to engaging in secure storage. The final three themes, also arising from five articles, addressed military-affiliated women’s perspectives on firearm discussions in the context of suicide prevention, including preferred or credible messengers for means safety information and perspectives and experiences with clinical interventions relating to lethal means safety.

#### 3.2.1. Theme 1: Women weigh safety considerations when making decisions about firearm ownership, access, and storage.

Decisions regarding firearm ownership, access, and storage were impacted by the unique experiences and needs of individuals. Concerns about safety were central to these decisions. For many women firearms were considered safety tools that enabled personal protection, while for others, firearms represented a threat to the safety of those living in the household. Notably, conceptions of safety shifted in response to personal and public events, altering ownership and storage decisions in response.

Motives for firearm ownership among women veterans are diverse, including hunting, sport, inheritance, collecting, and occupational needs. However, qualitative and quantitative studies both identified personal protection as a common and important factor motivating firearm ownership. Among 17 firearm-owning participants in one study, 16 reported owning a firearm for protection against strangers, nine listed protection from animals, and three reported protection from people they knew [38]. In survey of nearly 1000 women service members and veterans who had previously deployed, 21% reported keeping “guns or other weapons nearby in order to feel secure” after returning from deployment [39].

In interviews, participants described obtaining or keeping firearms for self-protection, often due to past experiences of interpersonal violence—such as military sexual trauma, intimate partner violence, harassment, threats, or discomfort in the male-dominated military environment [26,37,38]. Quantitative research supports these narratives with MST and PTSD symptom severity associated with increased engagement in firearm behaviors during the COVID-19 pandemic [26]. Similarly, lifetime sexual assault, lifetime domestic violence, and combat trauma exposure were all associated with higher odds of keeping a weapon nearby for safety following deployment [39].

Women who had experienced interpersonal violence described “never want[ing] to be put in those kind of situations again [37],” feeling better because they had “something to be able to protect [themselves] [37],” and “clinging to [their] firearm…[their] safety blanket [38].” Anxieties arising during the COVID-19 pandemic due to uncertainty and resource scarcity, social unrest and violence, and rising racial tensions also drove engagement with firearms [26]. One participant described the sentiment that “everyone else has one so it seems like I would need one too [26].” In many cases, the function of a firearm as a tool for personal protection influenced storage practices, with women often reporting storing firearms unlocked and loaded, keeping loaded firearms in easy to access locations, sleeping near a firearm, increasing access during periods of increased social conflict, or hesitancy to store firearms in a safe as they would be useless in an emergency [26,37].

While many women felt firearm accessibility was a crucial element of personal protection or safety, others felt safer limiting firearm access in their homes. In many cases, the same reasons that led some women to see firearms as tools for protection, led others to see them as potential threats. Mental health concerns and child safety were important motivators to either choose not to own firearms or restrict household access, especially when connected to suicidal ideation, histories of suicide attempts, and mental health concerns arising from MST or when young children were present [27,38,37]. Others got rid of firearms due to fears that owning one may escalate IPV-related risk, including harm to themselves or their children or the possibility of using it against an abusive spouse [37]. Finally, one woman raised concerns over the intersection of racial identity and firearm ownership and use as an African American woman, concluding that “nothing good could come of [her] using it” even for protection [26].

Psychological, interpersonal, and societal shifts, such as worsening mental health, family changes, or relationship stress, often prompted women to reconsider firearm ownership and storage [27,38,37]. Meanwhile, the increased instability related to civil unrest and racial tensions during the pandemic had the opposite effect, with fears over safety from the behaviors of others overcoming general concerns about safety and firearm accessibility [26].

#### 3.2.2. Theme 2: Military experiences and identity changed relationships with firearms.

Whether participants had prior experiences with and perspectives on firearms, their use in the military had a specific purpose – “protecting themselves from others who might hurt or kill them [37].” Some were uncomfortable around firearms prior to their service while for others firearm ownership was normalized in their communities, whether for hunting, sport, or protection [38,37]. In both groups, women described the importance of being able to properly use their firearm to kill in order protect themselves and their fellow service members from other people – “…it was a third arm [38],” “if you’re not familiar with it, then you aren’t any help to anyone [38],” “They are strictly there to kill people [38],” “It isn’t hunting anymore…. Now, I am being trained to shoot people [38],” “you’re willing to give up your life and protect others with this lethal means [38].” Beyond the specific purposes of firearm training in the military, some participants had personal motivations to obtain high levels of firearm expertise, including the desire to be perceived as equal in the male-dominated environment and to protect themselves from threats of harassment, threats, and assault [37].

#### 3.2.3. Theme 3: Relationship dynamics can impact firearm access and storage decisions.

Household firearm access was often facilitated by men, commonly by a male spouse or partner. In one qualitative study, over 40% of past and present firearm-owning participants had household access to a firearm controlled by someone else [38]. In some cases, partners purchased firearms for participants, including for shared hunting activities [37], for the participant to use for self-protection [37], or, because the male partner was legally barred from owning firearms [27]. Participants reported varying levels of knowledge and control over access and storage. While some couples engaged in collaborative storage decisions, others felt their preferences were dismissed by their partners [27,38,37]. For example, one participant described her husband purchasing firearms over her objections while she was deployed [37]. Another reported carrying a firearm bought by her spouse for protection despite not knowing how to use it [37]. In some cases, participants reported firearms being used as a tool to abuse or threaten, with the partner refusing to address safety concerns held by the participant [27].

#### 3.2.4. Theme 4: Lethal means safety interventions are generally perceived as effective and acceptable, but barriers to care remain.

Women veterans acknowledged the importance of LMSC and restricting firearms access as suicide prevention measures [38], with these considerations driving the personal decisions of women in restricting firearm access in their own homes and lives [27,38,37]. Additionally, when surveyed about the perceived effectiveness of a series of low intensity (screening, psychoeducation, and gun locks) and high intensity interventions (VA programs to assist with gun disposal, gun storage, or storage of keys to gun safes), all female participants endorsed at least one intervention, with over 85% endorsing at least one high intensity intervention [40].

Women veterans reported that lethal means conversations were acceptable in the context of suicide prevention. Circumstances included conversations alongside suicide risk assessments [38], during mental health crises [38,37], and even in the context of general mental health care [38,37], particularly if there was an established relationship with the provider [37]. Further, women veterans preferred mental health providers over medical providers as sources of means safety messages [37], while Black female service members identified organizations related to suicide prevention as the most credible means safety messengers [36].

While there was broad support for the effectiveness and acceptability of LMSC in the context of mental health care, barriers to and misconceptions of LMSC persisted for some. Some shared preferences for hearing from peers with military or law enforcement experience rather than providers [36], including desires for support groups specific to women veterans where means safety strategies could be shared [38]. Others raised concerns over the effectiveness and impacts of LMSC, including beliefs that suicide cannot be prevented [38], doubts about making recommended storage changes [37], and fears over the consequences of disclosing firearm access such as firearm seizure, involuntary hospitalization, and career impacts [38,37].

#### 3.2.5. Theme 5: Trust, caring, and established relationships were critical aspects of accepting LMSC.

While suicide prevention was perceived as an acceptable context for lethal means counseling, trust – in the institution and the provider – was a critical component of acceptability, receptivity, and effectiveness of individual instances of lethal means counseling. Health care settings and health care providers were rarely spontaneously mentioned as preferred messengers for lethal means counseling among women veterans [37]. In a survey of messenger credibility, white, active duty women tended to rank medical professionals lower than peers and shooting/firearm organizations [36]. Polzer, Holliday, and colleagues [38] identified institutional betrayal following help seeking for MST during service as a barrier to discussing firearms in VA settings. In another study, lack of trust and rapport with their clinician made two participants unsure if they would listen to recommendations because they might doubt the providers assessment of risk [37]. Similarly, one participant felt she could not share everything with a provider who had her hospitalized following a past suicide attempt [37]. Participants shared negative experiences in care that impacted receptivity to LMCS and perceptions of trustworthiness. These included demeanors that were cold, distant, or impersonal, approaches to firearm discussions that felt like a checklist (series of yes/no questions), and when they felt like the provider perceived them to be irresponsible or dangerous with their firearms [38].

Despite these concerns, participants broadly agreed that they would be more willing to discuss firearms and secure storage with a provider they had a longstanding, trusting relationship with [38,37]. Provider behaviors that helped to build trust included when the provider was “attentive, thoughtful, and demonstrating compassion and genuine care [[38], author quote],” when they were direct and clear about the reasons for the conversation, and when they actively listened without judgement [38]. Participants with histories of MST reported they felt more comfortable with female providers, while another shared that she was able to trust a male provider who respected her personal space during interactions [38]. Psychoeducation and reassurances of confidentiality were important aspects of building trust. Participants thought it was important for providers to address the threshold for firearm seizure and hospitalization and also to address the temporary nature of recommended means restriction during periods of elevated risk [38]. Finally, LMSC approaches that were grounded in military and firearm competency, emphasized concerns for the veteran’s general safety, and engaged with the veteran’s reasons for ownership promoted trust and rapport [38]. Essentially, the foundation of trusting relationships, and thus willingness to engage with LMSC, came down to quality clinical care [38]. Fostering trust at the provider level helped create comfortable atmospheres for LMSC, even in the context of institutional distrust [38].

#### 3.2.6. Theme 6: Spouses are key stakeholders in household firearm safety, but relationship dynamics influence their inclusion in care.

Spouses and partners played a central role in household firearm safety, with many women already discussing storage strategies, particularly in relationships characterized by trust and collaboration [27,38,37]. These conversations often addressed general safety, elevated mental health risks, or the presence of children in the home. Polzer, Rohs, and colleagues [27] identified three relational types based on the nature of firearm-related communication within relationships. In collaborative relationship types, both partners contributed to decisions about safe storage, sometimes engaging in detailed discussions of mental health and child safety [27].

Women in deferential relational types described trusting their partners to manage firearm storage independently but felt confident their concerns would be heard if raised. Conversely, in devalued relational types, often involving low trust and intimate partner violence, women reported that their safety concerns were dismissed or met with threats, sometimes involving the firearms themselves [27].

Despite these dynamics, family members—typically husbands—were sometimes identified as preferred messengers for firearm safety among firearm-owning women veterans [37]. One participant noted that open communication with her spouse would prompt her to reconsider her mental health and storage practices if he voiced concern. However, in a survey of active duty women, family members’ credibility as means safety messengers was rated only moderate to low [36], suggesting that while partners can play a powerful role in means safety for some, they are not universally trusted messengers.

## Discussion

In this systematic review, we synthesized research examining the perspectives and preferences of military-affiliated women on firearms and other lethal means, including views on ownership, access, use, storage, and suicide prevention interventions. Seven articles were identified, including three qualitative articles arising from two study populations, one mixed methods study, and three quantitative studies. All studies focused on firearm ownership and behaviors and/or firearm related screening and counseling, with six themes identified relating to perspectives on firearms and LMSC. Government-directed focus on suicide prevention and firearm safety campaigns have led to concerted research efforts over the past decade in efforts to bolster clinical care and interventions [41–43]. However, research relating to lethal means safety approaches for women veterans remains severely limited, despite rising suicide rates and elevated firearm-related suicide risk in this growing population.

Notably, this review failed to identify any literature relating to military and veteran women’s perspectives on non-firearm means safety. This gap echoes finding of other systematic reviews, which demonstrate significant literatures in areas relating to firearm means safety and restriction, limited publications relating to medication restrictions, and an absence of literature relating to other household means such as ligatures and sharps [17,44,45]. This emphasis on firearm safe storage is also reflected in military LMSC guidance [46–48], which provide minimal recommendations for medication restriction and, when acknowledged, generic advice to restrict access to other household means. The lack of research on perspectives of military-affiliated women on means safety strategies beyond firearms is particularly concerning given the elevated rates of completed and attempted suicides by strangulation and overdose in this community. This point is echoed in a recent review of the scope of LMSC delivered in EDs, which calls for additional support for non-firearm means safety given differences in suicidal behavior among different age groups and gender identities [44]. This finding demonstrates the critical need for additional research on stakeholder perspectives in this area.

Research relating to military and veteran women’s perspectives on firearms has increased over the last five years, following calls for additional attention to this population. Qualitative research in the review provided rich insights from veteran women relating to: their motivations for firearm ownership and storage decisions, experiences navigating firearm storage decisions with more (or less) concerned significant others, and their preferences and experiences with suicide prevention and lethal means counseling in the context of the VA [26,27,38,37]. Broadly, quantitative data supported findings from qualitative interviews relating to firearms as tools for personal protection [26,38,39] and effectiveness of means restriction interventions [40]. Women shared complex and nuanced perspectives on firearms that highlighted their importance as tools for personal protection, while acknowledging that firearms had the potential to harm those they sought to protect [27,37–39]. Perspectives on lethal means counseling in clinical settings were similarly nuanced. Participants expressed broad support for the effectiveness and acceptability of lethal means counseling in suicide prevention contexts but also shared significant apprehensions about discussing firearms in VA contexts due to experiences of institutional betrayal and fears relating to firearm seizure and involuntary hospitalization [26,27,38,36,37,40]. These findings point to several important clinical implications when conducting lethal means safety counseling with women veterans.

Institutional distrust and fears of involuntary hospitalization and firearm seizure are well documented barriers to disclosure of suicidal thoughts and behaviors and firearm access in military and veteran communities [49–52]. Provider perceptions of client barriers to engagement with LMSC and apprehensions surrounding responses may lead providers to avoid the subject [53]. In a study of recent VHA users, only 14% reported discussing firearm safety [54]. However, research among veterans and service members consistently demonstrates that firearm discussions are acceptable in the context of suicide prevention with trusted, culturally competent providers [50,51,55]. Trust was fostered through quality clinical care, including conveying genuine care and concern, actively listening without judgement, providing a rationale for LMSC focused on patient safety, educating around limits of confidentiality, and delivering culturally informed care informed by a basic understanding of firearm and military culture. These behaviors are frequently cited by service members and veterans as essential elements of suicide risk assessment and LMSC [51,52,55–58] and also recognized by mental health clinicians as strategies that promote trust and facilitate disclosure of suicidal thoughts and behaviors [59]. Women veterans also emphasized the importance of trauma-informed care, including providers’ respect for personal space, attention to the risks and impacts of intimate partner violence on means safety, and access to women’s groups and dedicated spaces, which helped alleviate concerns about harassment and service-related trauma while fostering connection with others who shared similar military experiences [38,37].

Women veterans experience elevated rates of MST and IPV [60] and sexual violence among women veterans has been linked to suicidal ideation and suicide attempts [14]. In a recent national cross-sectional survey, over half of veteran women experienced MST, nearly one-quarter experienced military sexual assault, and nearly half experienced lifetime IPV [60]. Histories of violence impacted participants in included studies at even higher rates, in some cases exceeding 80% [26,38,39], offering deep insights into the roles both MST and IPV play in firearm ownership and storage decisions and understandings of safety. While protection is consistently identified as a primary motivation for firearm ownership among veterans [21,61,62], articles reviewed here highlight the complexity of how veteran women conceptualize firearms, safety, and protection in the wake of histories of violence. For many participants with these experiences, safety means protection from external threats and sexual violence and necessitated quick access to their firearms [26,38,37]. For other participants, safety concerns centered around self-harm or accidental harm inflicted by a household firearm. Many women shared how mental health concerns, including those stemming from MST, past suicide attempts, and fears of escalating IPV led them to remove or securely store household firearms for their own protection [26,27,38,37]. Similar findings have been noted among women in the general US population, where histories of both sexual assault and IPV, particularly IPV involving firearms, motivated some women to own and carry firearms [63,64], while others reported fears that firearms may be used against them or be accessed by their children [64]. Diverging understandings of firearm safety between firearm owners and health care providers contribute to miscommunications that hamper harm reduction efforts [45,65]. It is important for clinicians to recognize and be prepared to address these differences, including psychoeducation around the relative risks of unsecured firearms in the home [66].

While hesitations persisted regarding firearm discussions in VA contexts, included studies demonstrate many women veterans support family and spousal involvement in lethal means safety conversations [27,38,37]. These findings follow evidence for widespread veteran support of family involvement in care, including safety planning for suicide prevention [67] and PTSD treatment [68,69], as well as findings from a recent systematic review of stakeholder perspectives on LMSC [45]. Qualitative research with spouses of women veterans offers preliminary evidence that partners are willing to help reduce firearm access but need additional support and guidance on actionable strategies to improve household firearm safety [70]. Conversely, many women reported resistance or dismissal when they attempted to discuss firearm storage with their spouses, who were often perpetrators of IPV [27]. While treatment modalities for IPV exist, they require relationship commitment, commitment to end relationship violence, and expertise on the part of clinicians [71]. Notably, these modalities are not appropriate in relationships where violence is used as a mechanism for intimidation and control [71], where access to firearms dramatically increases the risk of intimate partner homicide [72]. Decisions about involving family members in LMSC should carefully consider the relationship between the client and family member and should include assessment for IPV [27].

Rich qualitative insights provide a foundation for developing gender-informed means safety counseling for military-affiliated women, including a large mixed-methods project with qualitative survey responses from over 500 participants relating to firearm beliefs during the COVID-19 pandemic [26]. While the related quantitative research generally supports qualitative findings, most of the quantitative literature pre-dates the recent qualitative work. Future quantitative research building directly on qualitative insights is vital to identify broadly acceptable and effective lethal means safety interventions tailored to this elevated risk military subpopulation. Notably, nearly three-quarters of recent literature (2016–2023) relating to women veterans’ mental and physical wellness was funded by VA sources [73], with all qualitative research relating to military-affiliated women’s perspectives on lethal means conducted in a VA context with VA samples. While this research produces critical insights on mental health care and suicide prevention for women veterans, its focus fails to capture the perspectives and needs of active duty service members and the approximately 50% of women veterans who receive care outside of the VA due to either ineligibility or preference [74]. Given barriers to care around institutional mistrust, additional research on perspectives, needs, and opportunities for non-VA connected care are essential in developing holistic and comprehensive interventions for military-affiliated women.

Another key area for future research is the role of partners and other family or friends in lethal means safety interventions. Included studies demonstrated support for family involvement in LMSC among many participants [27,38,37]. However, research and guidance on how to best incorporate familial support into mental health care or engage support personnel outside of care remains limited. Moreover, many women shared circumstances where spouses introduced barriers to safe household firearm storage, ranging from dismissing safety concerns to using firearms as tools for intimidation and IPV [27,37]. Given the prevalence of IPV among women veterans, additional research is needed to identify circumstances in which it is appropriate to involve spouses in LMSC or to extend psychoeducation to them, and when partner involvement may further increase risk. In situations complicated by IPV, additional work is essential to develop means safety approaches that reduce firearm related suicide and homicide risk without escalating the potential for harm.

These review findings should be considered alongside several limitations, all of which highlight the limited scope of research in this area. Most notably, only seven articles met review criteria, with all qualitative analysis conducted by the same research team and two articles drawing from the same study participants. In addition, qualitative findings from three articles were based on small sample sizes and two quantitative studies had limited inclusion of women participants. Finally, while the diversity of research questions provides breadth to qualitative findings, it also limits thematic synthesis across a broader pool of participants. While generalizability is not the aim of qualitative research, the limited scope of research in this area points to the necessity of expanded research to better inform clinical practice.

This systematic review highlights the critical need for trauma-sensitive, gender-informed lethal means safety counseling tailored to military-affiliated women, a population which is at elevated risk for suicide but often overlooked in the peer reviewed literature [11,20]. Despite growing recognition of lethal means safety as a suicide prevention priority, research remains limited in scope, with few studies explicitly examining women’s perspectives. The few extant studies included in this review emphasize the complex relationship between personal protection, trauma history, MST/IPV, and trust in institutions, all of which impact perspectives on lethal means safety and firearm safe storage decisions. Future research must expand its focus beyond VA-based populations, to address the full range of military-affiliated women in the interest of enhanced understanding and generalizability. Future efforts should also consider and further elucidate the roles of family and intimate partners, including the risks posed by IPV and should pursue development of practical, acceptable, and effective strategies to serve this population. Finally, research must expand beyond firearms to include women’s perspectives on means safety for other means that are commonly involved in suicide deaths in this community. Through targeted clinical, research, and implementation strategies we can tailor means safety counseling and firearm safe storage interventions while fully acknowledging and contextualizing the lived experiences of military-affiliated women.

## Supporting information

S1 Appendix Search strategy. (DOCX)

S2 Appendix ENTREQ checklist. (DOCX)

S3 Appendix PRISMA checklist. (DOCX)
