# Supplementary material for: Perspectives of military-affiliated women on lethal means safety: A systematic review
Source: PLoS One. 2026 Mar 5;21(3):e0344104. doi: 10.1371/journal.pone.0344104 (PMC12962533; doi:10.1371/journal.pone.0344104)
Supplement: S1 Appendix — (DOCX) [file pone.0344104.s001.docx]

**S1 Appendix. Search strategy.**

*APA PsycINFO via Ovid*

1. (women* or woman* or female*).ti,ab,id.
2. Exp Human Females/
3. ((military* or army or "marine corps" or navy or naval OR "air force" or "space force" or "coast guard" OR “department of defense” OR “homeland security”) and (personnel or servicemember* OR airmen OR airwomen or serve* or officer* OR veteran* or soldier* OR troop* OR ranger* OR infantry* OR combat* OR guard*)).ti,ab,id.
4. Exp Military Personnel/ OR Exp Combat Experience/ OR Military Mental Health/ OR Military Psychology/ OR Military Families/ OR Military Measures/
5. (“Women veterans” OR “woman veteran”).ti,ab,id
6. ("lethal means" or suicid* or "murder-suicide" or "homicide-suicide" or "bridge barrier*" or gun or guns or firearm* or "safe storage" or "storage practices" or "self-injur*" or "self-harm*" or automutilat* or "self-destructive" OR “secure storage” OR crisis OR crises OR pills OR poison* OR ligature* OR “means safety” OR “means counsel*” OR “means restriction*” OR “safety counsel*” ).ti,ab,id.
7. Exp Self-Destructive Behavior/ OR firearms/ or gun violence/ OR prescription drug misuse/ or exp drug abuse/ or drug dependency/ or prescription drugs/ OR drug overdoses/
8. ((self or oneself or myself or themsel* or himself or herself) adj2 (kill* or harm* or injur* or hurt* or mutilat*)).ti,ab,id.
9. (Preference* OR experience* OR opinion* OR survey* OR counsel* Or treatment* OR risk* OR reduc* OR increas* OR decreas* OR prevent* OR interven* OR behavio* OR motivat* OR recommendation* OR resource* OR practices OR attitude* OR safety OR perspective*).ti,ab,id
10. Attitudes/ OR Exp Treatment/ OR Exp Risk Factors/ OR Exp Motivation/
11. (((1 OR 2) AND (3 OR 4)) OR 5) and (6 OR 7 OR 8) AND (9 OR 10)

*Ovid MEDLINE ALL*

1. (women* or woman* or female*).tw,kw
2. exp Women/ or (female/ AND exp humans/)
3. ((military* or army or "marine corps" or navy or naval OR "air force" or "space force" or "coast guard" OR “department of defense” OR “homeland security”) and (personnel or servicemember* OR airmen OR airwomen or serve* or officer* OR veteran* or soldier* OR troop* OR ranger* OR infantry* OR combat* OR guard*)).tw,kw
4. Military Personnel/ OR Veterans/ OR military deployment/ OR Military Family/
5. (“Women veterans” OR “woman veteran”).tw,kw
6. ("lethal means" or suicid* or "murder-suicide" or "homicide-suicide" or "bridge barrier*" or gun or guns or firearm* or "safe storage" or "storage practices" or "self-injur*" or "self-harm*" or automutilat* or "self-destructive" OR “secure storage” OR crisis OR crises OR pills OR poison* OR ligature* OR “means safety” OR “means counsel*” OR “means restriction*” OR “safety counsel*” ).tw,kw
7. exp Self-Injurious Behavior/ OR Firearms/ OR exp Gun Violence/ OR exp Substance-Related Disorders/ OR Prescription Drugs/
8. ((self or oneself or myself or themsel* or himself or herself) adj2 (kill* or harm* or injur* or hurt* or mutilat*)).tw,kw
9. (Preference* OR experience* OR opinion* OR survey* OR counsel* Or treatment* OR therapy OR risk* OR reduc* OR increas* OR decreas* OR prevent* OR interven* OR behavio* OR motivat* OR recommendation* OR resource* OR practices OR attitude* OR safety OR perspective*).tw,kw
10. exp Attitude/ OR exp Psychotherapy/ OR exp Psychiatry/ OR exp Risk Factors/ OR exp behavior/ or exp emotions/ or exp motivation/
11. (((1 OR 2) AND (3 OR 4)) OR 5) and (6 OR 7 OR 8) AND (9 OR 10)
12. remove duplicates from 11
